# The Enhancer of split complex arose prior to the diversification of schizophoran flies and is strongly conserved between *Drosophila *and stalk-eyed flies (Diopsidae)

**DOI:** 10.1186/1471-2148-11-354

**Published:** 2011-12-08

**Authors:** Richard H Baker, Jennifer V Kuehl, Gerald S Wilkinson

**Affiliations:** 1 Sackler Institute for Comparative Genomics, American Museum of Natural History, 79th at Central Park West, New York, NY 10024 USA; 2 Department of Energy Joint Genome Institute, 2800 Mitchell Drive, Walnut Creek, CA 94598 USA; 3 Physical Biosciences Division, Lawrence Berkeley National Laboratory 1 Cyclotron Rd Berkeley, CA 94729 USA; 4 Department of Biology, University of Maryland, College Park, MD 20742 USA

**Keywords:** Enhancer of split complex, stalk-eyed fly, gene duplication, SPS+A domain

## Abstract

**Background:**

In *Drosophila*, the Enhancer of split complex (E(spl)-C) comprises 11 bHLH and Bearded genes that function during Notch signaling to repress proneural identity in the developing peripheral nervous system. Comparison with other insects indicates that the basal state for Diptera is a single bHLH and Bearded homolog and that the expansion of the gene complex occurred in the lineage leading to *Drosophila*. However, comparative genomic data from other fly species that would elucidate the origin and sequence of gene duplication for the complex is lacking. Therefore, in order to examine the evolutionary history of the complex within Diptera, we reconstructed, using several fosmid clones, the entire E(spl)-complex in the stalk-eyed fly, *Teleopsis dalmanni *and collected additional homologs of E(spl)-C genes from searches of dipteran EST databases and the *Glossina morsitans *genome assembly.

**Results:**

Comparison of the *Teleopsis *E(spl)-C gene organization with *Drosophila *indicates complete conservation in gene number and orientation between the species except that *T. dalmanni *contains a duplicated copy of *E(spl)m5 *that is not present in *Drosophila*. Phylogenetic analysis of E(spl)-complex bHLH and Bearded genes for several dipteran species clearly demonstrates that all members of the complex were present prior to the diversification of schizophoran flies. Comparison of upstream regulatory elements and 3' UTR domains between the species also reveals strong conservation for many of the genes and identifies several novel characteristics of E(spl)-C regulatory evolution including the discovery of a previously unidentified, highly conserved SPS+A domain between *E(spl)mγ *and *E(spl)mβ*.

**Conclusion:**

Identifying the phylogenetic origin of E(spl)-C genes and their associated regulatory DNA is essential to understanding the functional significance of this well-studied gene complex. Results from this study provide numerous insights into the evolutionary history of the complex and will help refine the focus of studies examining the adaptive consequences of this gene expansion.

## Background

Expansion of gene families by gene duplication is a common feature of evolutionary history and is expected to provide a major source of novel genetic material needed to facilitate phenotypic evolution [1-5]. While most duplicates are rapidly lost from the genome, some are retained because of increased dosage requirements, the acquisition of new functions (e.g. neofunctionilization) or the splitting of the ancestral function between the duplicate copies (e.g. subfunctionilization) [5,6]. The genetic variation provided by gene duplication may be as important for adaptive evolution as replacement substitutions or changes in regulatory DNA [2,4]. Genomic-level comparisons that are now possible for closely related species in a few groups have provided fine-scaled resolution of shifts in gene family sizes and revealed that rapid changes in gene family composition is pervasive [3,7,8]. The evolutionary pressures shaping the size and structure of gene families can vary substantially in different lineages. For instance, an analysis of the 12 *Drosophila *genomes estimated that approximately 10% of all gene families are specific to a single lineage within the genus [1]. Precise mapping of the phylogenetic pattern of gains and losses in gene family structure and organization is necessary to understand the evolutionary factors driving these changes.

One gene complex that appears to be specific to *Drosophila *relative to other insects and may play an important role in the evolution of this genus is the Enhancer of split complex (E(spl)-C). This complex spans a 45 kb region in *Drosophila melanogaster *and comprises seven basic helix-loop-helix (bHLH) transcription factors (*mδ, mγ, mβ, m3, m5, m7, m8*), four Bearded (Brd) class genes (*mα, m2, m4, m6*) and a single gene (*m1*) thought to act as a protease inhibitor [9]. All the bHLH and Brd genes play a role in neurogenesis and function as negative regulators in the Notch signaling pathway [10-17]. Their primary role is to limit the number of progenitor cells during neural specification. For instance, in the formation of the adult peripheral nervous system, small clusters of cells acquire neural cell fate potential through the expression of proneural proteins such as Achaete and Scute. Only one of these cells, the Sensory Organ Precursor (SOP) cell, will develop into the components of the adult bristle. In response to Notch signaling, the E(spl)-C proteins specify the identity of the SOP by suppressing proneural protein expression in all cells adjacent to the SOP, a process known as lateral inhibition. Large deletions within the E(spl)-complex produce excessive neuronal differentiation [14,18], whereas elevated expression of the E(spl)-C proteins reduces sensory organ cells [15].

Despite the neural hyperplasia resulting from large deletions, it has been difficult to identify phenotypic defects caused by fine scale mutations within the complex and deletion of an entire gene is rarely lethal [17,19]. This pattern suggests strong functional redundancy among the genes [20]. Two other lines of evidence, however, indicate unique functional roles for each of the E(spl)-C genes. First, individual genes exhibit strong gene-specific expression patterns, particularly in the imaginal discs [10,21,22]. Second, comparisons between *D. melanogaster *and *D. hydei *indicate there have been no gene losses within the complex since the common ancestor of these species [20] suggesting that all of the genes are functionally important and maintained by stabilizing selection. Therefore, the expansion of the gene family may have been driven by selection pressures for greater complexity and specificity of N signaling in different tissues [23].

With respect to regulatory structure, the E(spl)-C genes are one of the best characterized loci in *Drosophila*. Although different members of the complex have distinct patterns of gene expression, they share many common features within their regulatory regions. The majority of genes in the cluster are regulated by Suppressor of Hairless (Su(H)) and several proneural genes. Numerous upstream cis-regulatory elements for these proteins have been identified [24,25]. One regulatory feature in particular, an inverted pair of Su(H) elements separated by 17 basepairs (bp) and in close association with a proneural binding site, appears to have strong functional significance. This regulatory architecture (termed a SPS+A element: Su(H) Paired Sites + proneural bHLH Activator binding site) resides upstream of many genes in the complex and it's relative location is strongly conserved among *Drosophila *species [24,25]. SPS+A elements have also been found in other non-dipteran insects and in other genes unrelated to the Enhancer of split genes [25]. Functional assays indicate the SPS+A element is a crucial component of the synergistic signaling response mediated by Su(H), proneural proteins, and several co-repressors and activators [26-29]. Regulation of the E(spl)-complex is also affected by a series of 3' UTR motifs that are bound by micro-RNAs (miRNAs) post-transcriptionally [30]. Similar to the cis-regulatory elements, these motifs occur in the majority of the E(spl)-C genes and exhibit strong conservation among *Drosophila *species [12,16,31,32].

The E(spl)-complex is unusual among gene expansions in that it involves the coordinated duplication of two different types of genes that have no close paralogy, but have functional overlap and share common regulatory mechanisms. Several recent studies have examined the evolutionary history of the complex [25,33,34] but have focused primarily on non-dipteran taxa. The mosquito species, *Anopheles gambiae *and *Aedes aegypti *each contain a single homolog of the bHLH and Brd genes suggesting the expansion occurred after the split between Nematocera and Brachycera. Comparison across the *Drosophila *genomes indicates the gene composition and much of the regulatory organization has remained stable since the emergence of this genus, approximately 40-60 MYA. However, little is known about the structure and regulatory content of the complex in dipteran species that represent intermediate evolutionary steps between Nematocera and *Drosophila*. This information is crucial to understanding the evolutionary history of the complex and the selection pressures influencing its expansion. Therefore, using sequence data from several fosmid clones from a genomic library, we reconstructed the entire complex in the acalyptrate stalk-eyed fly, *Teleopsis dalmanni*. In addition, we probed the recently sequenced genome of the tsetse fly, *Glossina morsitans*, along with other dipterans with a well-represented EST database, in order to reconstruct the history of the complex within schizophoran flies.

## Methods

### Study Organism

*Teleopsis dalmanni *is one of approximately 200 species in the acalyptrate family Diopsidae. All species in the family are characterized by the elongation of the head into long stalks and many species, including *T. dalmanni*, are sexually dimorphic with respect to their eyestalks. Annotation of expressed sequence tag (EST) libraries identified five contigs with significant homology to E(spl)-C genes in *Drosophila *[35] and Comparative Genomic Hybridization has placed all of these genes on one of the two autosomes of *T. dalmanni *[36]. The Acalyptratae is a large, derived group of flies that also contains the families Drosophilidae and Tephritidae. Relationships among acalyptrate families have proven difficult to resolve, but in several analyses the Tephritidae and Diopsidae are closely related and share a common ancestor with *Drosophila *no more than 76 MYA [37,38]. Alternatively, the Diopsidae were placed as the basal acalyptrate lineage in a recent study, but this analysis was limited to mitochondrial genes [39]. Overall, there is considerable debate concerning the monophyly of the Acalyptratae [37-41], but a general consensus that they are most closely related to the Calyptratae and together form the Schizophora. This group contains most of the well-studied dipteran species that do not belong to the Nematocera, comprise roughly half of the family-level diversity within Diptera, and are estimated to have diverged 80-100 MYA [37]. Some studies [38-40] have placed the Drosophilidae as closely related to the Calyptratae within a paraphyletic Acalyptratae.

### Fosmid Library Construction and Sequencing

A genomic library was constructed for *T. dalmanni *using the CopyControl Fosmid Library Construction Kit (Epicentre). These libraries accommodate inserts of approximately 40 kilobases. Genomic DNA was prepared from 90 developing flies dissected from their pupal case using a phenol/chloroform protocol previously applied to diopsids [42]. The flies were chosen from a large, outbred population of *T. dalmanni *originally collected in 1999 near Ulu Gombak in peninsular Malaysia and maintained at the University of Maryland. The prep provided nearly 60 μg of total DNA, of which 20 μg was used to construct the library. The genomic DNA was manually sheared using a syringe and ligated, packaged and plated following the manufacturer's protocol. Our library produced over 66,000 clones, thus providing approximately 4× coverage of the genome, which is estimated to be 450 MB. All colonies were picked from bioassay plates using Q-bot automated colony pickers (Genetix) and individually stored (at -70°) in 384-well plates. Restriction digest of 48 colonies using *KpnI *indicated all but one of the clones contained inserts between 30-40 kb.

Blast searches of ESTs generated from the developing eye-antennal imaginal disc of *T. dalmanni *[35] identified homologs of five members of the E(spl)-complex--*mβ, mα, m3, m4, m7*--in *Drosophila*. Based on these sequences, we designed primers for each gene (Additional file 1) in order to probe, using PCR, pooled aliquots of fosmid clones. Primers were also generated for *E(spl)m8 *after initial sequencing of a fosmid clone containing *E(spl)m7 *provided the nucleotide sequence for that gene. The pooling strategy we used combined all samples from a single 384-well plate into one target sample for PCR. A total of 171 plates were pooled in this fashion. If the PCR for a given plate produced a band for one of the E(spl)-C genes, we then pooled all the wells for each row and column of that plate for a second round of PCR. This round of PCR (involving 40 total reactions) identified the exact well location of the fosmid clone containing the E(spl)-C gene. The general reaction template of the PCR was 94°-2 m, (94°-30s, 52°-30s and 72°-45s)× 35 cycles, and 72°-7 m. Overall, PCR of the pooled fosmid plates identified 12 total fosmid clones containing E(spl)-C genes and seven of these were selected for sequencing. We generated a 3 kb sub-clone library for each selected fosmid by shearing the fosmid into ~ 3 kb fragments using a Hydroshear device (GeneMachines) and ligating the DNA into pUC18 vector. Colonies from the sub-cloned libraries were picked from bioassay plates using a Q-bot and arrayed in 384-well plates. All the clones from a single plate for each fosmid were sequenced in both directions providing approximately 10× coverage of the fosmid sequence. Sequencing of the sub-clones was conducted at the Joint Genome Institute (JGI) using their standard rolling-circle amplification protocol http://www.jgi.doe.gov/sequencing/protocols/prots_production.html. Quality scores for the sequencing reads for each fosmid were assigned to each base using Phred [43,44]. The reads were assembled using Phrap [45] and manually curated using Consed [46]. In a few cases, additional clones from a sheared fosmid library were sequenced in order to provide reads that spanned gaps between contigs in the assembly. Fosmid contigs were assembled into a larger genomic contig using Phrap and Sequencher (GeneCodes). The assembled contig has been submitted to Genbank under accession JN546230.

### Evolutionary Analysis

**Figure 1:**
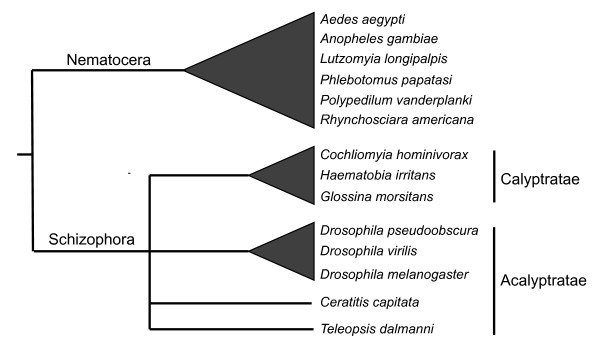
**Phylogenetic relationships among the dipteran species used in this study**. Calyptrate flies are thought to represent a monophyletic group but the relationships among acalyptrate flies are not well understood [37-41]. There is support for a close relationship between *Drosophila *and the Calyptratae within a paraphyletic Acalyptratae [38-40].

Identification and annotation of the transcription units within the *T. dalmanni *E(spl)-*C *genomic contig was conducted by blasting (with Blastx) the entire contig against the *D. melanogaster *NCBI protein database as well as aligning the *T. dalmanni *EST sequences to the genomic contig. We also performed syntenic comparison between the species using mVista [47,48]. In order to identify homologs of E(spl)-C genes in other dipteran species, we searched, using tBlastx, the NCBI EST database and *Glossina morsitans *supercontig database (http://www.sanger.ac.uk/cgi-bin/blast/submitblast/g_morsitans) for genes with strong similarity to *T. dalmanni *and *D. melanogaster *E(spl)-C genes. The *G. morsitans *sequence data were provided by the *Glossina morsitans *group at the Wellcome Trust Sanger Institute and can be obtained from maa@sanger.ac.uk. All ESTs with a Blast hit lower than e-20 were placed in a Sequencher folder and assembled together using a 95% identity similarity cut-off. Consensus sequences for each cluster were exported and translated into protein sequences. For the E(spl)-C bHLH and Brd genes, we aligned, using the Muscle alignment function [49] in the Geneious analysis package [50], the protein sequences from all E(spl)-C bHLH and Brd homologs for *Bombyx mori *(outgroup), *Anopheles gambiae*, *Aedes aegypti*, *G. morsitans*, *T. dalmanni*, *Drosophila virilis, Drosophila pseudoobscura*, *D. melanogaster *and seven other dipteran species identified in the EST search. These include the acalyptrate fruitfly *Ceratitis capitata*, two calyptrates--*Haematobia irritans *and the screw-worm fly *Cochliomyia hominivorax*--and four nematoceran species--*Lutzomyia longipalpis, Phlebotomus papatasi, Polypedilum vanderplanki *and *Rhynchosciara americana*. Phylogenetic relationships among these species are presented in Figure 1. A maximum likelihood tree was constructed from these aligned matrices (Additional file 2) in PhyML [51] using a WAG+G model with 100 bootstrap replicates. Pairwise non-synonymous to synonymous substitution ratios were calculated using PAL2NAL [52].

In order to identify conserved regulatory domains, we searched all non-coding DNA within the E(spl)-C complexes of *T. dalmanni *and *G. morsitans *for Su(H) binding sites (YGTGRGAA) and proneural A boxes (RCAGSTG) [24]. An inverted pair of Su(H) sites with an A box in close proximity constitutes the SPS+A architecture found in many Drosophila E(spl)-C genes. If this domain was found in any of the *T. dalmanni *or *G. morsitans *genes, we extracted 200 bp of sequence data on either side of the domain and aligned it to the region containing the homologous SPS+A domain in *Drosophila *using Dialign [53]. We also searched the 3' UTR regions for the three conserved domains in *Drosophila *known to influence post-transcriptional regulation [30-32]: the Brd box (AGCTTTA), GY box (GTCTTCC), and K box (TGTGAT). For genes for which we had EST sequence (in either *T. dalmanni *or other non-*Drosophila *dipterans) the 3' UTR region was determined by the transcript sequence. For *T. dalmanni *and *G. morsitans *genes for which there was genomic sequence data, but not transcript sequence, we searched 1000 bp 3' of the stop codon for that gene.

## Results

### The E(spl)-Complex in *Teleopsis dalmanni *and *Glossina morsitans*

**Figure 2:**
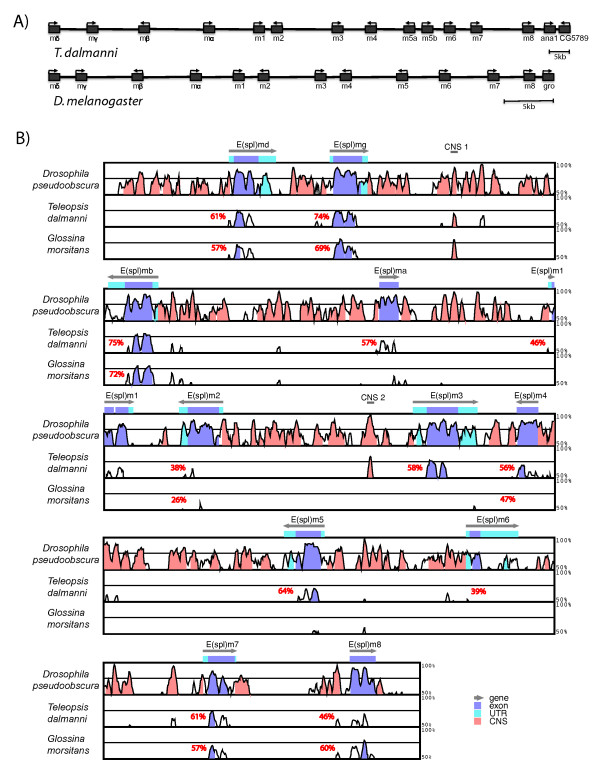
**Genomic organization of *T. dalmanni *E(spl)-complex**. (A) Gene location and orientation is provided for all E(spl)-C genes in *T. dalmanni *and *D. melanogaster*. (B) Genomic alignment of the E(spl)-complex in *D. pseudoobscura*, *T. dalmanni and G. morsitans *to the E(spl)-complex in *D. melanogaster*. Average pairwise amino acid similarities relative to *D. melanogaster *are provided for each E(spl)-C gene in *T. dalmanni *and *G. morsitans*

The seven *T. dalmanni *fosmids sequenced in this study assembled into one primary contig spanning 145 kb that contained a direct homolog of each of the E(spl)-C genes found in *Drosophila*. Although spread out across a larger region in *T. dalmanni *than *D. melanogaster*, the organization of the complex in terms of gene order and orientation is identical between the species (Figure 2a). One difference between the species is that *T. dalmanni *has an additional gene homologous to *E(spl)m5 *in *Drosophila *suggesting a recent duplication within stalk-eyed flies. Pairwise comparison of synonymous and non-synonymous substitution rates between the duplicate copies (dN/dS = 0.088) did not indicate any signs of positive selection operating on the genes following duplication. Another difference between the species is that, in *Drosophila*, the E(spl)-complex is adjacent to *groucho*, a gene that is a Notch-mediated co-repressor of E(spl)-C genes [54] and that has often been included as a member of the complex [20,24]. In *T. dalmanni*, however, the E(spl)-complex is adjacent to genes that are homologous to *anastral spindle 1 *(ana1) and CG5789 in *D. melanogaster*. These two genes are adjacent to each other and located on chromosome 3R within 200 kb of the E(spl)-complex in *D. melanogaster*. The percent identity scores for individual E(spl)-C genes between *T. dalmanni *and *D. melanogaster *range from 75% (*E(spl)mβ*) to 48% (*E(spl)m8*) among the bHLH genes and from 57% (*E(spl)m*α) to 39% (*E(spl)m6*) for the Brd genes (Figure 2b). There is little similarity in the non-coding sequence data between the species although there is a small region of conserved sequence data between *E(spl)mγ *and *E(spl)mβ *(CNS1 in Figure 2b) and upstream of *E(spl)m3 *(CNS2 in Figure 2b).

Homologs for most of the E(spl)-C genes (*mδ, mγ, mβ, m2, m4, m7, m8*) were also found in *G. morsitans *indicating that the expansion of the gene complex is ancestral to the evolution of the Schizophora. All these genes occurred in the same order and orientation as in *D. melanogaster *and *T. dalmanni*. We were unable to locate in the *G. morsitans *genomic contigs a full length homolog for *E(spl)m*α, *E(spl)m1*, *E(spl)m3*, *E(spl)m5*, and *E(spl)m6*. Both *E(spl)m1 *and *E(spl)m6 *are rapidly evolving genes which may affect our ability to identify the genes in *G. morsitans*, although a clear homolog of *E(spl)m1 *(identity: 45%, blast score: 2e-34) was found in the horn fly *Haematobia irritans *(Muscidae) suggesting the gene was present in the acalyptrate-calyptrate ancestor. In the *G. morsitans *assembly, the intersection between two supercontigs (0000482 and 0005687) spans the region where *E(spl)m5 *should be located and one of the contigs (0005687) contains a fragment with partial similarity to *E(spl)m5 *genes in *D. melanogaster *(e-07) and *T. dalmanni *(e-12). This fragment, however, lies near the 5' region of the gene and there is no upstream start codon within the fragment's open reading frame. Similarly, *E(spl)mα *in *G. morsitans *is only a partial fragment of the gene but there is a string of Ns in the supercontig (0000482) so the absence of the remaining portion may result from an error in the contig assembly. Additional sequence data is necessary to determine if *E(spl)m*a and *E(spl)m5 *are functioning genes in *G. morsitans*.

### Evolution of E(spl)-complex within Diptera

**Figure 3:**
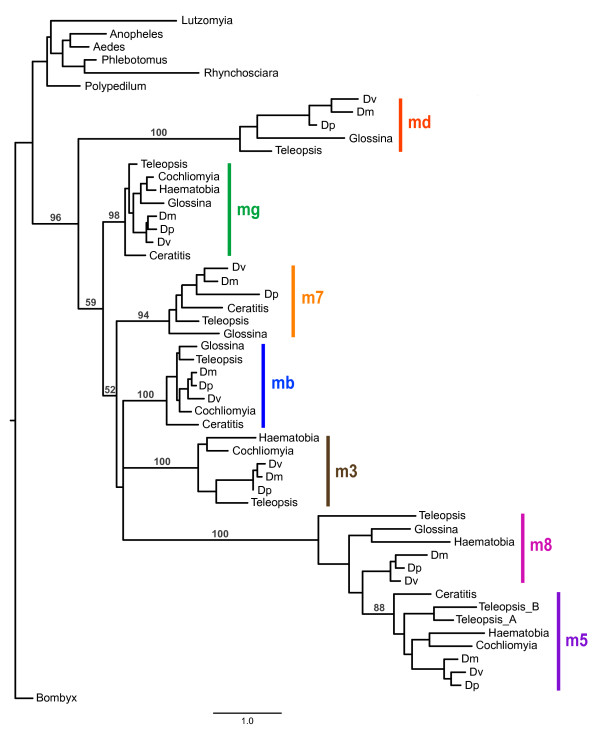
**Phylogenetic analysis of bHLH E(spl)-C genes in Diptera**. Bootstrap values greater than 50 are provided for nodes defining and joining paralogs. Dm *- D. melanogaster*, Dp *- D. pseudoobscura*, Dv - *D. virilis*.

In order to examine the evolutionary history of the complex within Diptera we obtained all E(spl)-C homologs from several dipteran species whose genomes have been sequenced and from seven additional species for which EST data was available (Figure 1). Sequence from the silkmoth *Bombyx mori *was used as the outgroup. Phylogenetic analysis of all the bHLH genes aligned together is presented in Figure 3. This tree clearly demonstrates that the expansion of the E(spl)-complex occurred after the split between Nematocera-Brachycera and before the diversification of the Schizophora. All the nematoceran species contain a single E(spl)-C bHLH homolog that cluster in a monophyletic clade that is basal to the other taxa. Each of the clades representing the individual E(spl)-C genes is well supported (bootstraps > 94%) and contains at least one calyptrate and acalyptrate species. It is important to note that absence of homologs for *Ceratitis*, *Haematobia*, and *Cochliomyia *(e.g. *E(spl)mδ*) probably reflects the limitations of transcript sampling from EST studies and not loss of these genes in these taxa.

Despite the clear differentiation among the individual E(spl)-C genes, the relationships among them are not well supported. Therefore, it is difficult to reconstruct the pattern of gene duplication among the various copies. *E(spl)m8 *and *E(spl)m5 *likely descended from a single duplication event because homologs from these genes comprise a strongly supported clade (100% bootstrap) but the pattern among the other genes suggests that the expansion process was characterized by rapid diversification followed by relative stasis. The phylogeny also confirms the duplication event for *E(spl)m5 *in *T. dalmanni *and further suggests that *E(spl)m3 *was lost in *G. morsitans *because the two other calyptrates sampled in the tree (*Haematobia *and *Cochliomyia*) contain homologs of this gene.

**Figure 4:**
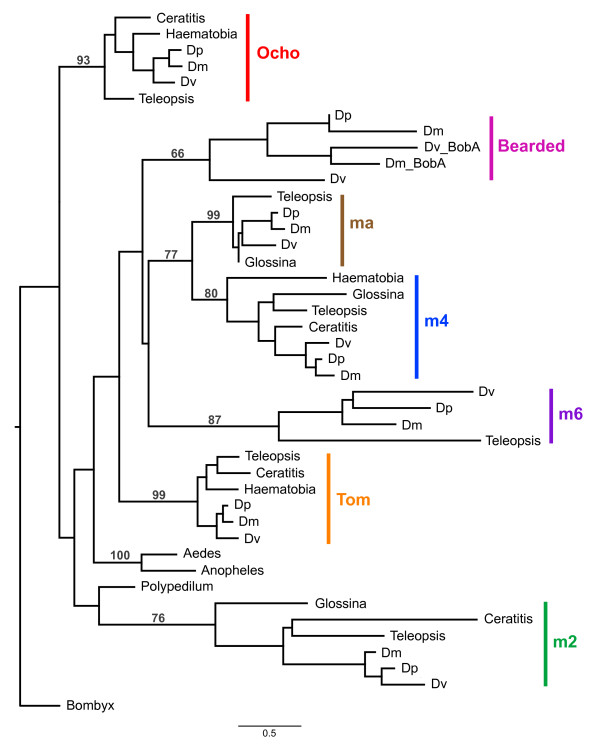
**Phylogenetic analysis of Bearded E(spl)-C genes in Diptera**. Bootstrap values greater than 50 are provided for nodes defining and joining paralogs. Dm *- D. melanogaster*, Dp *- D. pseudoobscura*, Dv - *D. virilis*. BobA *- Brother of Bearded A*.

The phylogeny for the Brd genes is presented in Figure 4. Closely related Brd genes that are not members of the E(spl)-complex [16,30]--*Tom*, *Ocho*, *Bearded*--were included in the analysis. Similar to the bHLH analysis, all the genes form relatively well-supported monophyletic clades and, with the exception of *Bearded *and *E(spl)m6*, contain at least one calyptrate and acalyptrate species. The relationships among the genes is not well supported although there is some support (77% bootstrap) for a sister relationship between *E(spl)mα *and *E(spl)m4*. In addition, the gene family as a whole is not monophyletic in this tree *(Ocho and E(spl)m4 *are sister to the nematoceran species rather than their paralogs in other schizophoran species) but these relationships are supported by bootstraps less than 10%.

### Conservation of SPS+A binding sites

**Figure 5:**
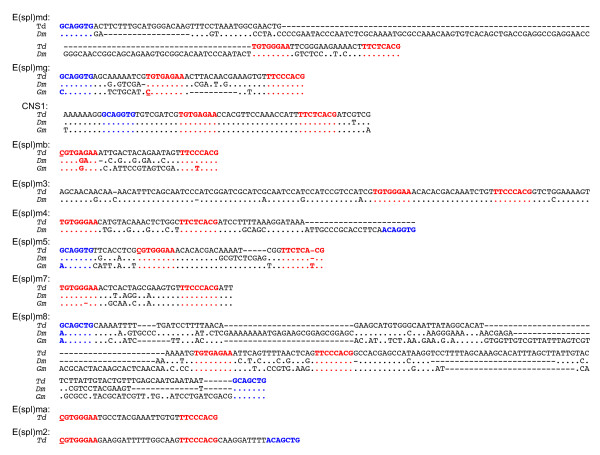
**Alignment of SPS+A regulatory modules**. Regions in red indicate 'S' binding domains and regions in blue indicate 'A' binding domains. Nucleotide sites at the beginning of the 5' 'S' domain that contain a C, rather than the canonical T, are underlined. Td - *T. dalmanni*, Dm *- D. melanogaster*, Gm *- G. morsitans*.

An inverted pair of Su(H) binding sites spaced 17 bp apart with a proneural activation site (A box) in close proximity constitutes the SPS+A regulatory architecture that has been found in the upstream regulatory sequence of numerous E(spl)-C genes and that plays an important role in the regulatory control of these genes [24,27,28,55]. Some genes have slight variants on this architecture involving either smaller spacing between the Su(H) site or the lack of a proneural box near the Su(H) pair. A search of the *T. dalmanni *and *G. morsitans *contigs for both Su(H) sites and proneural boxes identified several domains conserved between these species and *D. melanogaster *(Figure 5). Overall, *T. dalmanni *contained seven SPS+A domains and four SPS pairs without an associated proneural box and there was stronger conservation between *T. dalmanni *and *D. melanogaster *than between either species and *G. morsitans*. Perhaps the most noteworthy SPS+A domain was located in a highly conserved stretch of 64 bp between *E(spl)mγ and E(spl)mβ *(CNS1 in Figure 2 and 5). This region has nearly perfect identity among the three fly species and, to our knowledge, has not been identified in previous surveys of *Drosophila*, presumably because it is located downstream of both *E(spl)mγ and E(spl)mβ*. This is the only domain in *G. morsitans *that exhibits the canonical SPS+A regulatory code and the extreme conservation among the species suggests it serves an important functional role. The one region of regulatory DNA that exhibits greater conservation between *T. dalmanni *and *D. melanogaster *than CNS 1 is the SPS domain of *E(spl)m3 *(CNS2 in Figure 2 and 5). This 110 bp region has 93.6% identity between the species and complete identity in the spacer region separating the Su(H) pairs. There is no associated A box in this conserved region, or near the SPS pair in either species. Neither the protein coding gene nor the upstream regulatory region for *E(spl)m3 *were found in *G. morsitans*.

In addition to the CNS1 location, *E(spl)mδ, E(spl)mγ, E(spl)m7 *and *E(spl)m8 *contain SPS+A domains conserved between *T. dalmanni *and *D. melanogaster*. In *T. dalmanni*, the *E(spl)mδ, E(spl)mγ, E(spl)m7 *and *E(spl)m8 *domains are located 4970 bp, 553 bp, 469 bp and 338 bp respectively, upstream of the gene's start codon compared to 1419 bp, 353 bp, 761 bp, and 275 bp in *D. melanogaster*. The pair of Su(H) sites in *E(spl)mδ *is separated by 15 bp rather than the typical 17 bp (Figure 5), a feature also shared by *Drosophila *species [24,25]. The length conservation between *T. dalmanni *and *D. melanogaster *suggest this spacing has functional significance and is under stabilizing selection. *G. morsitans *does not appear to contain a SPS architecture in *E(spl)mδ *and, *for E(spl)mγ*, there is an inverted pair of Su(H) sites but they are separated by only 7 bp. For *E(spl)m7*, both *T. dalmanni *and *D. melanogaster *have a pair of Su(H) sites with a relatively distant A box (232 bp in *T. dalmanni *and 405 bp in *D. melanogaster*). *G. morsitans *has a mutation (a deletion) in the first Su(H) binding site that violates the consensus sequence and no A box between the Su(H) site and the start of the gene. All three species have a conserved SPS+A module (with two A boxes on either side of the Su(H) pair) in *E(spl)m8. E(spl)m4 *has a SPS module conserved between *T. dalmanni *and *D*. melanogaster but the A box present in *D. melanogaster *is missing from *T. dalmanni*. In *T. dalmanni*, because of the inverted orientation of the *E(spl)m5 *duplication, a single SPS+A domain lies upstream of both genes.

The SPS+A configuration was originally defined as not only having the inverted pairs of Su(H) sites separated by 17 bp, but also having a T in the "Y" position of the upstream Su(H) site and a C in this position in the downstream site [13,24]. Two *Drosophila *genes--*E(spl)mβ *and *E(spl)m5*--contained SPS pairs that have a C in the "Y" position of the upstream site but they also have spacer regions that differ from the typical 17 bp suggesting these domains may not be fully functional SPS+A pairs and that the C resulted from relaxed functional constraints [24,25]. One noteworthy feature of the *T. dalmanni *enhancer elements is the high occurrence of paired Su(H) sites with the canonical 17 bp spacing that have a C in the "Y" position of the upstream Su(H) sites. Four genes--*E(spl)mβ*, *E(spl)mα*, *E(spl)m2 *and *E(spl)m5--*have SPS modules with this nucleotide sequence and two of these--*E(spl)m2 *and *E(spl)m5--*also have an associated A box (Figure 5). This pattern suggests that the T nucleotide in the "Y" position of the upstream site may not be a requirement of a fully functional SPS+A module. This result is consistent with a recent study in humans showing that SPS elements with sequence degeneracy relative to the canonical structure can still drive expression [56].

### Conservation of 3' UTR regulatory boxes

**Figure 6:**
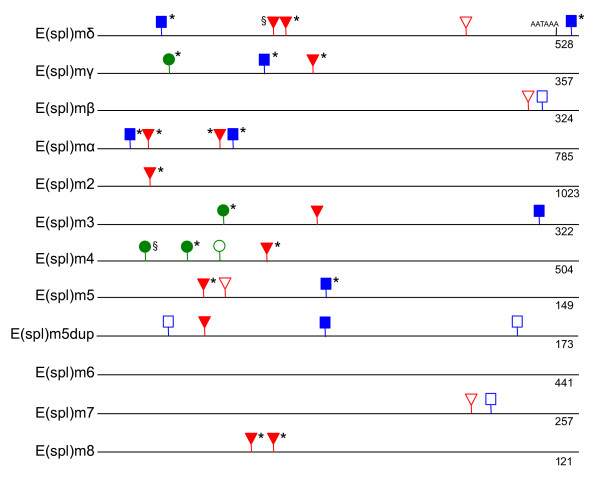
**Organization of 3' UTR domains in *T. dalmanni *E(spl)-C genes**. As in [30], GY-boxes are represented by green circles, Brd-boxes by blue squares and K-boxes by red triangles. Shaded shapes are conserved between *T. dalmanni *and *D. melanogaster*. Asterisks indicate domains that are found in one or more calyptrate species. § indicates domains not present in any calyptrate species for which there is available sequence information. Numbers under the lines provide the size (bp) of the fragment represented in the figure.

Post-transcriptional regulation of both bHLH and Brd E(spl)-C genes is mediated by a series of 3' UTR binding domains that are targeted by miRNAs [25-27]. Comparison of the 3' UTR domain structure between *T. dalmanni *and *D. melanogaster *(Figure 6) indicates remarkable conservation over the 60-100 million years separating the species. Five of the 11 genes (and six of 11 if we include *E(spl)m6*, which has no domains in either species) are completely conserved with respect to the number and organization of domains. Four of these--*E(spl)mγ*, *E(spl)mα*, *E(spl)m2 *and *E(spl)m8--*are completely conserved between acalyptrate and calyptrate species while the fifth gene, *E(spl)m3*, may be conserved between these groups but the available calyptrate EST, from *H. irritans*, contains only 100 bp of nucleotide sequence downstream of the conserved GY box (Figure 6). Three of the genes that are not fully conserved across the species--*E(spl)mδ, E(spl)m4*, and *E(spl)m5--*still have several domains in common. *E(spl)mβ *and *E(spl)m7 *are the two genes that exhibit no similarity in 3' UTR regulatory structure between *T. dalmanni *and *D. melanogaster*.

## Discussion

### Evolution of E(spl)-complex gene structure and organization

Despite widespread study of the developmental genetics of the E(spl)-complex in *Drosophila *[10-17], there is little consensus about the evolutionary pressures responsible for the origin and maintenance of this gene expansion. Overall, the genes appear to be partially redundant such that they can compensate for the loss of one member of the complex, but they also all have gene-specific expression patterns suggesting redundancy alone is not sufficient to explain their evolution [10,21,22]. It has been proposed that the presence of efficient post-transcription regulation mediated by the interaction between the 3' UTR domains and miRNAs facilitated duplication by minimizing the impact of deleterious gain-of-function effects that are likely to result from the duplication events [31]. This hypothesis provides a mechanism for the initial stability of the gene expansion but does not explain the fixation of the various copies or the selection pressures that maintain their evolutionary conservation. Understanding the functional role of this complex in *Drosophila *provides only partial information needed to explain its evolution. It is also necessary to identify when the various gene expansions occurred and what phenotypic modifications arose concurrently with the duplication events. In this study, we begin to address this issue by reconstructing the E(spl)-complex in a stalk-eyed fly and identifying homologs for every member of the complex among several calyptrate species. These data clearly establish that the E(spl)-complex expanded in entirety at or before the origin of the schizophoran lineage and has exhibited remarkable conservation since that time.

At three levels of gene organization--amino acids, promoter Su(H) binding sites, and 3' UTR domains--there is strong conservation among the orthologs from different species but little hierarchical signal among the paralogous copies. There are numerous molecular features that identify a gene sequence from a given species as belonging to a specific E(spl)-C gene, but less information about how these genes are related to each other. This weak phylogenetic signal among the members of the complex suggests that the expansion occurred rapidly and it is possible that we will find no species that have an intermediate stage of the expansion for the complex. This pattern of punctuated duplication [57], does not appear to fit a classic birth/death model of gene expansion [58], although we do not currently have sufficient sampling of the entire complex from enough species to evaluate this statistically. However, it is clear there have been no changes in the complex during the 40-60 MY since the formation of *Drosophila *and going back to the base of the Schizophora we only have evidence of the loss of *E(spl)m3 *in *G. morsitans *and the gain of an additional copy of *E(spl)m5 *in *T. dalmanni*. It is important to note that the lack of additional copies of a given E(spl)-C gene for taxa with EST data (that would indicate lineage specific duplication) is not an artifact of our search methodology because in nearly all cases a Blast search using a single E(spl)-C protein from *T. dalmanni *or *D. melanogaster *was sufficient to return all the paralogs of a given type (i.e. bHLH or Brd) that were available for a species. For instance, blasting with *E(spl)mγ *returned hits for all five bHLH genes of *C. hominivorax *included in Figure 3.

Some studies [25,34] have argued that *E(spl)mβ *is the original bHLH gene (and *E(spl)mα *is the original Brd gene) because it has the highest amino acid similarity to the single copy bHLH gene in Nematocera and the orientation of *E(spl)mβ *and *E(spl)mα *mirrors the bHLH and Brd gene orientation in Nematocera. The orientation is suggestive of an orthologous relationship but protein similarity is not necessarily indicative of a basal status. There are numerous reasons, such as relaxed stabilizing selection or multiple duplications and divergence operating on the original source paralog, to explain why the original copy would not have the highest protein similarity to genes in species without the duplicated copies. In our tree, *E(spl)mδ *is the basal gene in the tree but this should not be taken as evidence that *E(spl)mδ *is the direct ortholog of the nematoceran gene. First, the relationships among the genes in the tree are not well supported so it is difficult to be confident in the basal position of *E(spl)mδ*. Second, even if the tree was well supported, a basal position on the tree only indicates that *E(spl)mδ *was part of the original duplication event not that it is the direct ortholog of the nematoceran bHLH gene. For instance, the tree is consistent with a scenario in which *E(spl)m3 *is the original copy, duplicates and produce *E(spl)mδ*, duplicates again to produce *E(spl)mγ *and so on, as the source of all the duplication events other than the *E(spl)m5 *- *E(spl)m8 *split. Unless a species is discovered that has two bHLH and two Brd genes (i.e. represents the initial duplication events) it is unlikely we will unambiguously identify the original E(spl)-C genes.

Regardless of the origination source, the diversification and subsequent stability of the E(spl)-C genes suggest they have strong functional significance. The evolution of schizophoran flies is characterized by an increase in the stereotype patterning of large sensory bristles on the notum called macrochaetes [59]. These structures are absent from nematoceran flies, but are arranged in various array patterns in the Schizophora [59-61]. Given the phylogenetic origin of the E(spl)-complex demonstrated in this study and the role of these genes in bristle formation, it is tempting to speculate that selection pressures related to the sensory input provided by macrochaetes was a primary factor driving the diversification of genes in this complex. The Achaete-Scute complex, which contains the genes directly controlling bristle development in the SOP and which are repressed by E(spl)-complex genes in adjacent cells, is also thought to have diversified by a series of gene duplications in the dipteran lineage leading to *Drosophila *after the split with the Nematocera [34,62]. Therefore, it is essential for additional studies to probe the genomes and transcriptomes of several orthorraphous brachyceran species that are phylogenetically intermediate between the Nematocera and Schizophora. Given sufficient taxonomic sampling, we might be able to uncover an interspecific correlation between the evolution of bristle morphology and the origin and diversification of gene content and regulatory structure within the E(spl)-complex. Attempts to connect phenotypic variation with genetic variation at E(spl)-C loci at the intraspecific level have proven to be difficult [63,64], so a comparative approach may be more fruitful.

### Evolution of E(spl)-complex regulatory DNA

The E(spl)-complex represents one of the most well characterized regulatory systems in *Drosophila *and functional analysis has highlighted the importance of upstream SPS+A architecture in the regulation of E(spl)-C genes [11,27-29,65]. Examination of the SPS+A organization in the *Teleopsis *E(spl)-complex revealed strong conservation with *Drosophila *indicating the functional significance of these modules. The regulatory sequence of *Teleopsis *also points to some novel features of the SPS+A organization including the identification of a highly conserved SPS+A module in both species (CNS 1) that has not previously been identified in *Drosophila*. E(spl)-C genes are noteworthy for the proximity of the enhancer elements that regulate their gene expression to the promoter sites of these genes. Transgenic constructs comprising relatively small regions of regulatory sequences (.5 - 2 kb) are generally sufficient to recapitulate gene-specific expression patterns [10]. Given this organization, the identification of the strongly conserved SPS+A module downstream of both *E(spl)mγ *and *E(spl)mβ *is unexpected. That this module also exhibits complete conservation at the nucleotide level in the regions spanning the two S binding sites and the A binding site across *Drosophila*, *Teleopsis *and *Glossina *(Figure 5) suggests that it plays a critical role in the regulation of E(spl)-C genes. Functional studies in *Drosophila *are necessary to determine whether this module affects the expression of one or multiple genes within the complex. In addition, sequence comparison of SPS+A modules across multiple *Drosophila *species revealed elevated levels of nucleotide conservation in the regions between and adjacent to the paired S sites for several E(spl)-C genes [25]. This pattern suggests these nucleotides may serve some functional role beyond providing proper spacing between binding sites. Because of its extreme conservation, the SPS+A module identified in CNS1 provides an ideal experimental system to investigate the regulatory significance of this DNA and its potential impact on species-level expression differences.

In addition to CNS1, the *Teleopsis *sequence revealed two additional paired S sites, upstream of *E(spl)mα *and *E(spl)m2*, that are not present in *Drosophila*, with the latter belonging to the SPS+A class (Figure 5). Both genes contain single upstream S sites in *Drosophila*. Sampling of E(spl)-C regulatory sequence from additional taxa will be necessary to determine whether the paired orientation was gained in the lineage leading to *Teleopsis *or lost in *Drosophila*. A recent study in *Drosophila *[29] showed that experimental manipulation of SPS+A regulatory organization from a paired to a single S site, and vice versa, can reverse the transcriptional dynamics of genes downstream of these sites. The proneural gene *achaete *contains a single S site and expression of the gene is repressed in the presence of *Notch *signaling. When the regulatory organization is altered to contain a paired S site module, transcription is activated in the presence of *Notch *signaling. Conversely, *E(spl)m8*, which contains the SPS+A module and is normally activated by *Notch*, is repressed by Notch when one of the S sites is removed. Therefore, understanding the precise evolutionary sequence of gains and losses in S binding domains is critical for interpreting the functional significance of E(spl)-C regulatory architecture in *Drosophila*.

As with the SPS modules, there is strong conservation of 3' UTR domain organization between *Teleopsis *and *Drosophila*. Several genes, such as *E(spl)mγ, E(spl)mα, E(spl)m3 *and *E(spl)m8*, have diverged very little since the split between calyptrate and acalyptrate flies but others, such as *E(spl)m4 and E(spl)m7*, have several lineage-specific domains [30]. How these differences in the evolutionary stability of 3' UTR DNA correlates with phenotypic variation is unclear. Despite a wealth of research in the past decade on the biology of miRNAs that bind to these UTR domains (see [66,67] for reviews), little is known about the functional consequences of variation in domain organization. This limitation, combined with a lack of expression data for E(spl)-C genes in any fly species other than *D. melanogaster*, makes it difficult to speculate on the evolutionary significance of differences in 3' UTR DNA across species and E(spl)-C paralogs. A recent study in *C. elegans *has demonstrated that multiple UTR domains from a single gene group together into discrete modules that operate in a combinatorial manner to repress gene expression [68]. It is possible that a similar process exists for the E(spl)-C genes, but additional comparative studies on the 3' UTR domain structure and the expression patterns of different genes in various tissues are needed to uncover the regulatory logic utilized by this complex.

## Conclusions

The E(spl) complex in *Drosophila *comprises several bHLH and Bearded genes that function in neurogenesis as negative regulators of the Notch signaling pathway. Comparison with mosquitoes indicates the complex arose after the split between nematoceran and brachyceran dipterans but details on the precise pattern of gene family expansion remains unclear. Here, we reconstruct the entire complex in the acalyptrate stalk-eyed fly, *Teleopsis dalmanni*, and combine this data with EST and genomic sequence data from several other species to demonstrate that the complex arose in entirety prior to the diversification of schizophoran flies. Phylogenetic relationships among the various paralogs in both gene families suggest the history of the complex is characterized by rapid duplication and diversification followed by relative stasis. Strong conservation is also evident among both the 5' and 3' regulatory domains. Comparison of non-coding E(spl)-C DNA between *Teleopsis *and *Drosophila *revealed a previously unidentified, highly conserved SPS+A domain between *E(spl)mγ *and *E(spl)mβ *that presumably has strong functional significance, as well as other canonical SPS domains not present in *Drosophila*. The pattern of gene expansion for the E(spl) complex is consistent with a role in the evolution of stereotypical macrochaete bristle patterning but additional studies are needed to demonstrate a clear association between E(spl)-C diversification and bristle evolution.

## Authors' contributions

RB conceived of the study, constructed the genomic library, carried out data analysis and drafted the manuscript. JK prepared the fosmid clones for sequencing, conducted contig assembly and carried out experiments to close gaps between contigs. GW conceived of the study and drafted the manuscript. All authors read and approved the final manuscript.

## Supplementary Material

Additional file 1 ***T. dalmanni *E(spl)-C primers**. Primers of the E(spl)-C genes used to probe the pooled samples of the *T. dalmanni *genomic library. Click here for file

Additional file 2 **Protein alignment of bHLH and Bearded genes**. Alignment file of the E(spl)-C proteins for all dipteran species used to generate phylogenies in Figure 3. and Figure 4. Click here for file
