# Supplementary material for: The Enhancer of split complex arose prior to the diversification of schizophoran flies and is strongly conserved between Drosophila and stalk-eyed flies (Diopsidae)
Source: BMC Evol Biol. 2011 Dec 8;11:354. doi: 10.1186/1471-2148-11-354 (PMC3261227; doi:10.1186/1471-2148-11-354)
Supplement: Additional file 1 — T. dalmanni E(spl)-C primers. Primers of the E(spl)-C genes used to probe the pooled samples of the T. dalmanni genomic library. [file 1471-2148-11-354-S1.DOC]

Td E(spl)Mb F: GAGCACATGAAAAAGCTACGTGC

Td E(spl)Mb R: CTTGCAGATAATTTAAACGATGACC

Td E(spl)Ma F: AGATTTGTTGCAGCCAGCTT

Td E(spl)Ma R: CAGAAATCACCCCATCTGCT

Td E(spl)M4 F: GAAATGGAAGATTATGAATACGG

Td E(spl)M4 R: GTGGTACAGCAATTTGATTTGCGG

Td E(spl)M3 F:GAGGGTGAGCATGTAACACG

Td E(spl)M3 R:TGCGATATTTGGTCTGCAGC

Td E(spl)M7 F: GTATGCAGCAGCAGTGTGGT

Td E(spl)M7 R: ATCCGCTTGAGACTGGACTG

Td E(spl)M8 F: GCAGAATGCAGCGATAATGA

Td E(spl)M8 R: GGCGACTTTGTTGATTTGGT
